# Supplementary material for: Monitoring of Measurable Residual Disease Using Circulating DNA after Allogeneic Hematopoietic Cell Transplantation
Source: Cancers (Basel). 2022 Jul 7;14(14):3307. doi: 10.3390/cancers14143307 (PMC9323743; doi:10.3390/cancers14143307)
Supplement: Supplementary file 1 [file cancers-14-03307-s001.zip › cancers-1737893-supplementary.pdf]

cancers

MDPI

Supplementary Materials

Monitoring of Measurable Residual Disease Using Circulating DNA after Allogeneic Hematopoietic Cell Transplantation

Miguel Waterhouse, Sandra Pennisi, Dietmar Pfeifer, Florian Scherer, Robert Zeiser, Justus Duyster, Hartmut Bertz, Jürgen Finke and Jesús Duque-Afonso

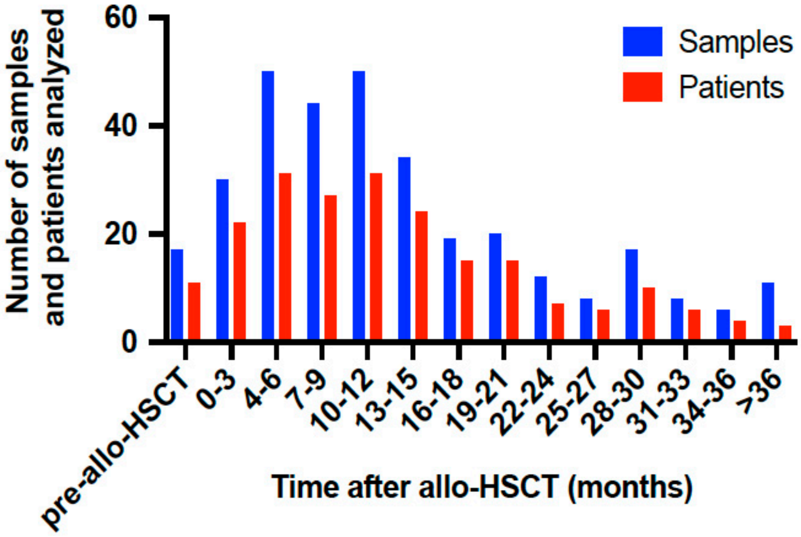

Figure S1. Number of samples, including chimerism and measurable residual disease, and patients analyzed at different time points after allo-HSCT. Allo-HSCT, allogeneic hematopoietic stem cell transplantation.

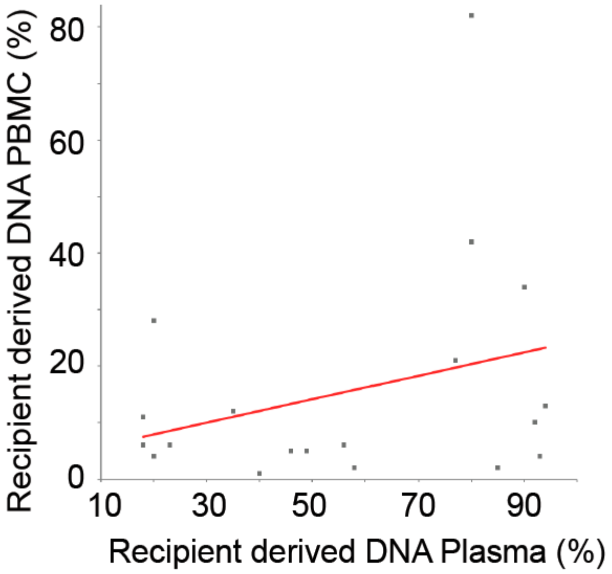

Figure S2. Determination of the optimal discriminating threshold of mixed donor chimerism in cfDNA to detect hematological relapse. (A) Correlation between percentage of recipient DNA in paired plasma and PBMCs samples. No significant correlation was observed (Spearman r=0.148). cfDNA, cell-free DNA; PBMCs, peripheral blood mononuclear cells.

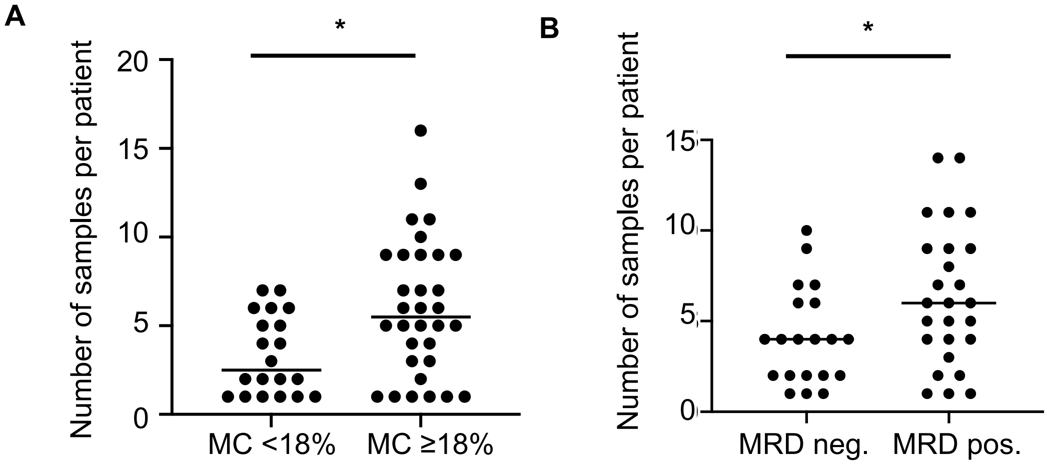

Figure S3. Number of samples per patient analyzed for MC and MRD in cfDNA. Number of samples per patient in cfDNA were analyzed by **(A)** mixed chimerism above or below the predetermined cut-off of 18% and **(B)** MRD positivity. MC, mixed chimerism; MRD, measurable residual disease; * p-value <0.05 by Mann-Whitney test.

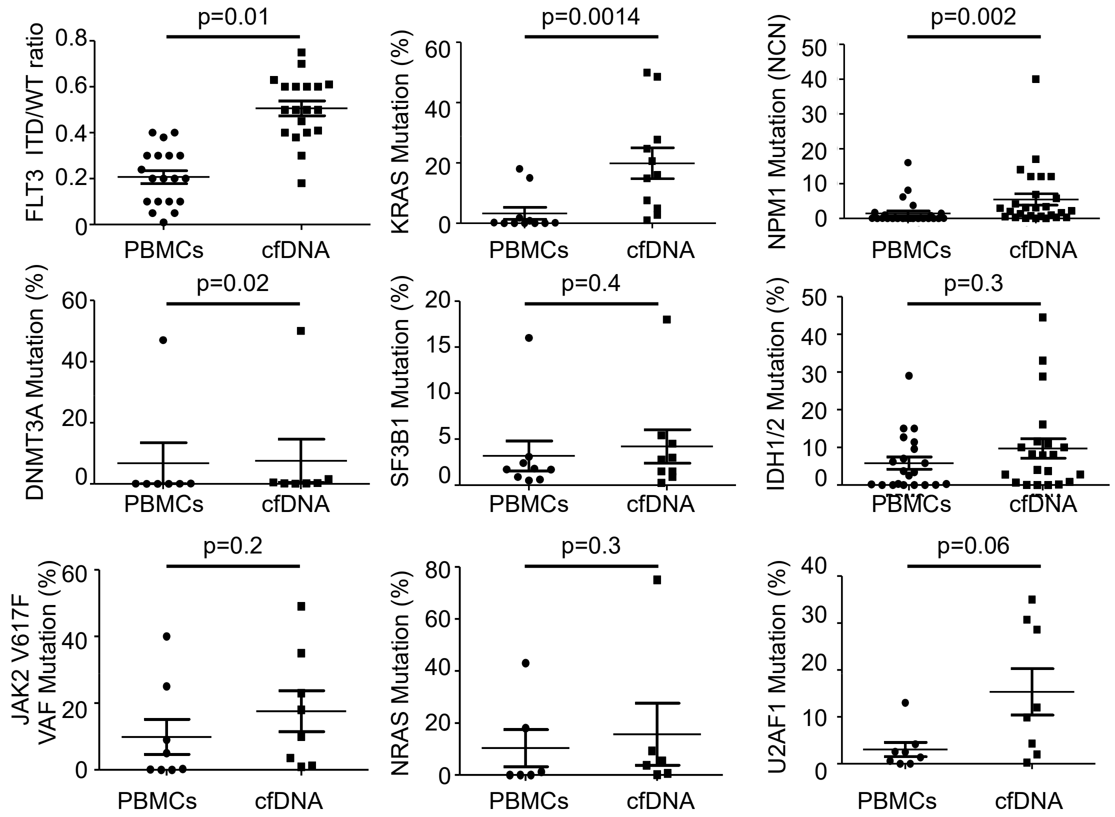

Figure S4. Representation of mutation tumor load PBMCs and cfDNA for each specific gene. Each graph represents a gene. Each dot represents a sample of a patient with the specific mutation. KRAS, DNMT3A, SF1B3, IDH1/2, JAK2, NRAS and U2AF1 are expressed as percentage of the mutation. NPM1 is expressed as normalized copy number. FLT3-ITD is expressed as ratio: ITD/WT. PBMCs, peripheral blood mononuclear cells; cfDNA, circulating-free DNA. Statistical analysis was performed by Mann-Whitney test.

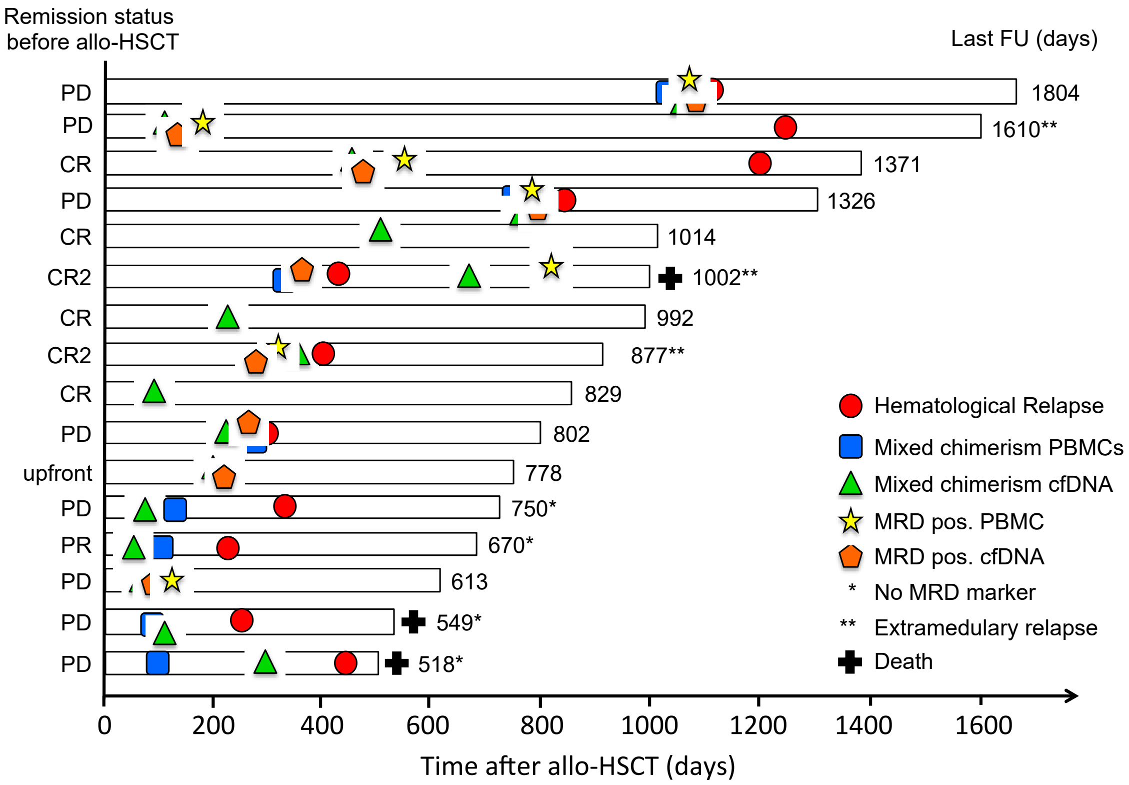

Figure S5. Detection of mixed chimerism and minimal residual disease in PBMCs and cfDNA as a function of time in selected patients. First detection of mixed chimerism and minimal residual disease in PBMC and cfDNA was plotted from selected patients in a time line after allo-HSCT. Hematological relapse, extramedullary relapse and death of patients is also depicted. In several patients, mixed chimerism and MRD were detected in cfDNA before in PBMCs. MC, mixed chimerism; MRD, minimal residual disease; PBMC, peripheral blood mononuclear cells; cfDNA, circulating-free DNA; allo-HSCT, allogeneic stem cell transplantation; pos., positive; CR, complete remission; CR2, 2. Complete remission; PR, partial remission; PD, progressive disease.

Table S1. Correlation analysis in cfDNA between mutation load or minimal residual diease) and recipient derived cfDNA (mixed chimerism). *NRAS, KRAS, IDH2 and SF3B1 are expressed as percentage, FLT3-ITD is expressed as ratio=ITD/FLT3 wild type, NPM1 is expressed as normalized copy number (NCN). cfDNA, circulating cell-free DNA; rs, correlation factor.

MRD Mutation	Mean Mutation Load in cfDNA *	Mean Recipient derived cfDNA (%)	Correlation (rs)
NRAS	3.5	31.2	0.4
NPM1	3.9	25.8	-0.6
KRAS	17	72.4	0.5
IDH2	15.5	42.5	0.99
FLT3-ITD	0.49	85.3	0.026
SF3B1	10.5	20.1	0.7
